# The role of concurrent chemoradiotherapy in the treatment of locoregionally advanced nasopharyngeal carcinoma among endemic population: a meta-analysis of the phase iii randomized trials

**DOI:** 10.1186/1471-2407-10-558

**Published:** 2010-10-15

**Authors:** Li Zhang, Chong Zhao, Bijesh Ghimire, Ming-Huang Hong, Qing Liu, Yang Zhang, Ying Guo, Yi-Jun Huang, Zhong-Zhen Guan

**Affiliations:** 1 Department of Medical Oncology, Sun Yat-Sen University Cancer Center, 651 Dongfeng Road East, Guangzhou, China; 2 Department of Radiation Oncology, Sun Yat-Sen University Cancer Center, 651 Dongfeng Road East, Guangzhou, China; 3 Clinical Trials Center, Sun Yat-Sen University Cancer Center, State Key Laboratory of Oncology in Southern China, 651 Dongfeng Road East, Guangzhou, China; 4 Department of Pharmacology, Medical School of Sun Yat-Sen University, Guangzhou, China

## Abstract

**Background:**

The main objective of this meta-analysis was to determine the clinical benefit of concurrent chemoradiotherapy (CCRT) compared with radiation alone (RT) in the treatment of nasopharyngeal carcinoma (NPC) patients in endemic geographic areas.

**Methods:**

Using a prospective meta-analysis protocol, two independent investigators reviewed the publications and extracted the data. Published randomized controlled trials (RCTs) in which patients with NPC in endemic areas were randomly assigned to receive CCRT or RT alone were included.

**Results:**

Seven trials (totally 1608 patients) were eligible. Risk ratios (RRs) of 0.63 (95% CI, 0.50 to 0.80), 0.76 (95% CI, 0.61 to 0.93) and 0.74 (95% CI, 0.62 to 0.89) were observed for 2, 3 and 5 years OS respectively in favor of the CCRT group. The RRs were larger than that detected in the previously reported meta-analyses (including both endemic and non-endemic), indicating that the relative benefit of survival was smaller than what considered before.

**Conclusions:**

This is the first meta-analysis of CCRT vs. RT alone in NPC treatment which included studies only done in endemic area. The results confirmed that CCRT was more beneficial compared with RT alone. However, the relative benefit of CCRT in endemic population might be less than that from previous meta-analyses.

## Background

Nasopharyngeal carcinoma (NPC) is a common malignant disease of the head and neck with a high prevalence in Southern China and Southeast Asia. It is different from other head and neck cancers because of unique epidemiology, natural behavior and therapeutic considerations.

NPC is both a radiosensitive and chemosensitive tumor. Since the publication of the results of a multicentre randomized trial conducted in North America (Intergroup study 0099) [1], concurrent chemoradiotherapy (CCRT) has been accepted as standard in the treatment of patients with stage III and IV NPC gradually. However, the major concern remains in extrapolating the findings of the intergroup study to patient groups in the Asian context, where NPC is endemic. Several meta-analyses and a pooled data analysis [2-5] had shown an improvement of survival in NPC patients who received chemotherapy and radiotherapy (CR+RT) versus those received radiotherapy alone (RT). Unfortunately, it still remains unclear regarding the benefit of CCRT especially for endemic population in the previously published meta-analyses. This fact is all these meta-analyses included heterogeneous histological mix of patients, limited number of studies published, or complexity of study design (CCRT with or without adjuvant or neoadjuvant chemotherapy versus RT alone). In contrast, a number of clinical studies [6-18] mainly focus on the additional value of CCRT from endemic areas has been published in recent years.

To gain a better understanding of the potential benefit of CCRT in endemic population, we undertook a meta-analysis that pooled data from all published Phase III randomized controlled trials (RCTs) done in endemic areas focusing on the impact of CCRT comparing with RT alone on patients with locally advanced NPC. To our knowledge, this is the first meta-analysis that included only those randomized trials done in endemic areas to date. The pooled analysis of largest cohort (1608 patients) should provide a clearer understanding of the impact of CCRT on the natural history of this disease.

## Methods

A prospective meta-analysis protocol including study aim, study selection criteria, literature search strategy, quality control of literature and statistical procedures was developed. The primary aim of present analysis was designed to evaluate how the CCRT influenced survival at 2, 3 and 5 years after treatment compared with RT alone in endemic area patients with locally advanced NPC. More specifically, the analysis was designed to examine the difference in patterns of failure (locoregional recurrence, distant metastasis) in CCRT and RT alone treatment group. In addition to the main meta-analysis, we also compared the survival difference between the CCRT with/without adjuvant chemotherapy (AC) and RT alone.

### Study Criteria

The selection criteria for eligible studies in this meta-analysis included published randomized controlled trials done in endemic area recruiting NPC patients of Asian origin. Patients were randomly assigned to receive radiotherapy alone or concurrent chemotherapy combined with radiotherapy. Patients receiving concurrent chemotherapy plus some form of adjuvant chemotherapy in addition to radiotherapy were also included in this analysis. The 1997 UICC TNM staging system was used for the staging of the primary tumor. CT or MRI was used as the main evaluation method and adequate doses of radiotherapy was given in both arms equivalent to at least 64Gy, with conventional fractionation to the primary lesion. Overall survival (OS) was the primary outcome measure for measuring the effect of treatment.

### Literature Search Strategy

The meta-analysis aimed to include all the relevant published trials done in endemic areas. To conduct a search of the published literatures, multiple search tools by two independent investigators were used: 1.Computerized bibliographic databases: Electronic databases (MEDLINE, CANCERLIT, and EMBASE) were searched with the medical headlines such as Nasopharyngeal carcinoma, Concurrent chemoradiotherapy, radiotherapy, and randomized controlled trials, to identify potentially eligible trials. 2. Journal manual search and reference lists: The computer search was supplemented with manual search of reference lists of all available review articles, primary studies, abstracts from meetings, and bibliographies of books. 3. Conference proceedings of ASCO, ESMO/ECCO, ASTRO and ESTRO.

**Figure 1:**
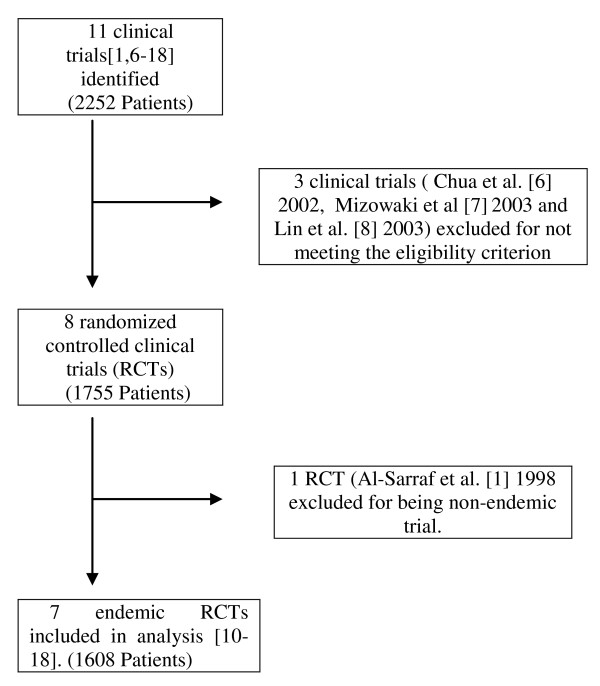
**A flow chart showing the selection of the trials**.

11 clinical trials including 2252 patients were initially identified. Among them, 4 trials were secondarily considered ineligible for different reasons (Figure 1) [1,6-18]. In total, 4 trials were excluded. The trial reported by Al-Sarraf et al [1] was excluded because this randomized trial was conducted in North America. The trials by Chua DT et al [6] and Mizowaki et al [7] were excluded in this meta-analysis for these trials not being randomized trials. The trial by Lin et al [8,9] was excluded for lack of samples size calculation and introduction of randomization method. It was also excluded by another earlier meta-analysis by Bertrand Baujat [5] for not meeting the eligibility criterion of unpredictable treatment assignment.

### Application of literature quality

The trials were reviewed using a list of predefined pertinent issues that concerned the characteristics of patients and treatments. To assess the methodological quality of RCTs, we have examined the statistical design, the randomization process, the quality control process, the dropout rate and if potential bias was taken into account. All analyses followed the intention-to-treat principle. This meta-analysis was performed according to preferred reporting items for systematic reviews and meta-analyses - The PRISMA statement [19].

### Method of data retrieves

Two independent investigators reviewed the publications and extracted the data. The following information was extracted from each article: 1. Basic information from papers such as, year of publication, journal name, and author name etc. 2. Characteristics of patients such as: age, sex, pathological types, stage, and study duration. 3. Information of study designation such as: sample size per group, study design, randomization scheme, inclusion criteria, and type of end point used. 4. Information of treatment such as: treatment modality, dose of RT, response rate of treatment, numbers of death, locoregional recurrence, distant metastasis, withdrawals, and so on. Available information was extracted and recorded to a data collection form and entered into electronic database.

### Statistical Analysis

The primary end point of this meta-analysis was OS, defined as the time from random assignment to death. Secondary end points were the incidence of local and/or regional recurrence (LRR) and distant metastasis (DM). Extraction of summary statistics from the Kaplan-Meier curve was performed according to standard methods for survival endpoints proposed by Parmar et al [20]. Standard techniques for meta-analysis were used [21].

Results were expressed as relative risk (RR) with 95% confidence intervals (CIs). The RR of survival at 2, 3, and 5 years and RR of LRR or DM at 3 years were compared between the CCRT and RT alone groups. RR less than 1 indicated improved survival for the combined-modality treatment compared with radiotherapy alone. When the span of the 95% CI given did not include 1, the result was statistically significant. Before estimation of a RR, a statistical test for homogeneity was performed. A Dersimonian and Laird random effects model [22] was used in cases in which statistically significant heterogeneity between studies likely existed. If no significant heterogeneity was found, a fixed-effects model was used to calculate pooled RR and 95% CIs. All analyses were conducted using Review Manager Version 5.0.24 (Revman; the Cochrane Collaboration; Oxford, England).

## Results

### Study identification and eligibility

After the selection procedure (Figure 1), 7 trials were considered eligible [10-18]. The majority of patients were included after 1990. The characteristics of these studies are listed in Table 1. Kwong's trial is a factorially designed study to test the efficacy of CCRT and adjuvant chemotherapy (AC) independently [10]. Patients were divided into four treatment groups: Group A, RT alone; Group B, CCRT; Group C, RT and AC; Group D, CCRT and AC. As in our analysis, we only took those randomized studies, which included comparison between CCRT vs. RT or CCRT +AC vs. RT. We did not include the patients in group C for the analysis. Subsequently for OS and overall locoregional and distance failure, group A was compared with group B and D. For subgroup analysis with pure CCRT vs. RT, groups A and B were compared and with adjuvant groups A and D were compared.

**Table 1:** Summary of studies included in the meta-analysis

Study	No. of patients	Inclusion period	Group	Histology (WHO grade, No.)			Stage	Radiotherapy	Chemotherapy	
				**I**	**II**	**III**			**Concurrent**	**Adjuvant**

Kwong et al, [10] 2004	165	1995-2001	CCRT RT	1 1	14 4	95 50	AJCC stage II -IV, any T, any N	2.5GyFx/5days/wk, primary site- 68Gy, Nodes- 66Gy, + 10Gy boost dose were given for pharyngeal extension and residual nodes	UFT 200 mg/day/7 days a wk	Alternating Cisplatin 100 mg/m^2 ^day1 and 5FU 1 gm/m^2^/d day 1-3 and VBM regimen (Vincristine 2 mg, bleomycin 30 mg, MTX 150 mg/m^2^) every 3wks for 6 cycles.

Chan et al, [11] 2005	350	1994-1997	CCRT RT	2 1	12 7	160 168	AJCC stage II to IV, any T, any N, M0	66Gy in 33Fx per 6.5 wks + additional boost in case of parapharyngeal extension, residual neck nodes, and/or residual nasopharyngeal disease (Brachytherapy)	Cisplatin 40 mg/m^2 ^in day1 weekly	None

Wee et al, [12] 2005	221	1997-2003	CCRT RT	-	100% grade II and III		AJCC stage II to IV, any T, any N	70Gy (2Gy/d in 5Fx/wk for 7 wks)	CDDP 25 mg/m^2^/d for 4 days, alternatively 30/30/40 mg/m^2^/d for 3 days if patient starts RT on Wednesday	CDDP 20 mg/m^2^/d × 4 days, 5FU 1000 mg/m^2^/d × 4 days

Lee et al, [13,18] 2005,2010	348	1999-2004	CCRT RT	-	100% grade II		AJCC stage III and IV, any T, N2 or N3, M0	≥66Gy (2Gy/Fx/d, 5Fx/wk) + additional boosts to the parapharyngeal space, the primary or nodal sites when indicated not exceeding 20Gy	Cisplatin 100 mg/m^2 ^× 3wks on days 1,22,43	CDDP 80 mg/m^2 ^and 5FU 1000 mg/m^2^/d every 4 wks on days 71,99 and 127

Zhang et al, [14] 2005	115	2001-2003	CCRT RT	-	100% grade II and III		AJCC stage III and IV, any T, N2 or N3, M0	70-74Gy (2Gy/Fx/d, 5fx/wk) + additional boost in case of parapharyngeal extension, residual neck nodes and/or residual nasopharyngeal disease	6× Oxaliplatin 70 mg/m^2 ^weekly	None
Lee et al, [15,17] 2006,2009	93	1999-2004	CCRT RT	-	100% grade II		AJCC stage III and IV, T3-4, N0-1, M0	≥66Gy (2Gy/Fx/d, 5Fx/wk) + Additional boosts to the parapharyngeal space, the primary or nodal sites when indicated not exceeding 20Gy	Cisplatin 100 mg/m^2 ^× 3wks on days 1,22,43	Cisplatin 80 mg/m^2 ^and 5FU 1000 mg/m^2^/d on days 71,99 and 127
Chen et al, [16] 2008	316	2002-2005	CCRT RT	-	100% grade II and III		AJCC stage III and IVA-B, T1-4, N0-3,	≥68Gy (2Gy/Fx/d, 5Fx/wk) in 7 weeks + additional boost in case of parapharyngeal extension, residual neck nodes and/or residual nasopharyngeal disease	Cisplatin 40 mg/m^2 ^day1 weekly × 7wks	Cisplatin 80 mg/m^2 ^day1 and 5FU 800 mg/m^2^/d on days1-5 every 4wks for 3 cycles.

Abbreviation: CDDP, Cisplatin; UICC, International Union Against Cancer; AJCC, American Joint Committee on cancer; FU, Fluorouracil

All 7 trials [10-18] were pooled together and 1608 patients were randomly assigned; of whom 773 received RT and 835 received combined modality treatment. For 3 years OS, the 6 trials [10-15] were included. Out of 6 studies, in three studies [10,11,14] including 573 patients, CCRT were compared with RT alone whereas in four studies [10,12,13,15] including 774 patients, AC was added to CCRT.

### Overall survival

**Figure 2:**
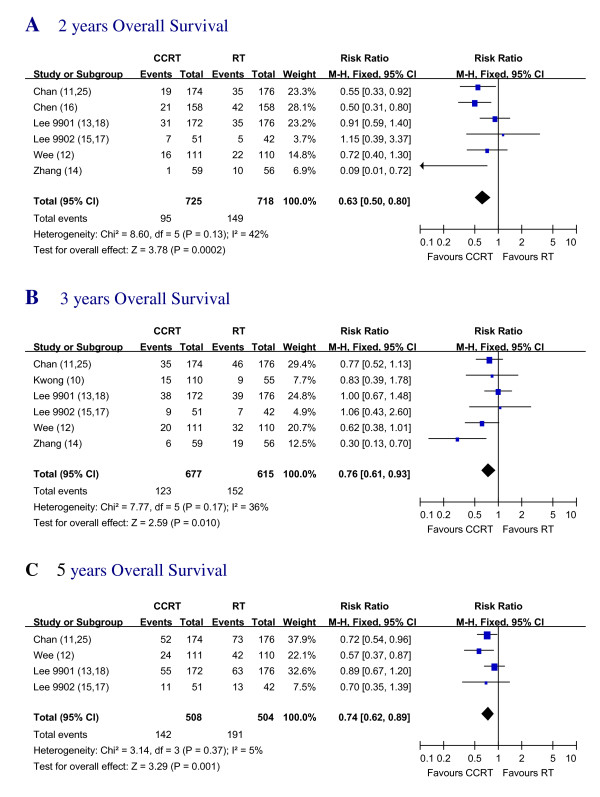
**Two, Three and Five years Overall Survival of CCRT vs. RT**. Forest plot of the risk ratio (RR) of the 2 years, 3 years and 5 years OS with radiotherapy (RT) alone versus radiotherapy plus concurrent chemotherapy (CCRT). The estimate of the RR of each individual trial corresponds to the middle of squares and the horizontal line gives 95% CI. The closed diamond shows the overall RR with its 95%CI. RR less than 1 indicated improved survival for the CCRT compared with RT alone.

Data regarding the OS of all the 7 trials [10-18] were available. The trial by Kwong et al [10] was not included in 2 years and 5 years OS calculations due to insufficient data. The trial by Chen et al [16] was not included in 3 years and 5 years OS calculations for too early to get 3 and 5-year data. Our own trial (Zhang et al [14]) was not included in 5 years OS calculations also for too early to get 5 years data. When 2-year overall survival rates were calculated, it showed significant benefit in favor of the CCRT treatment with RR of 0.63 (95% CI, 0.50 to 0.80). 3-year OS also showed significant benefit in favor of the CCRT treatment with RR of 0.76 (95% CI, 0.61 to 0.93). 5 years OS was significantly better in favor of the CCRT treatment groups with an RR of 0.74 (95% CI, 0.62 to 0.89) (Figure.2).

**Figure 3:**
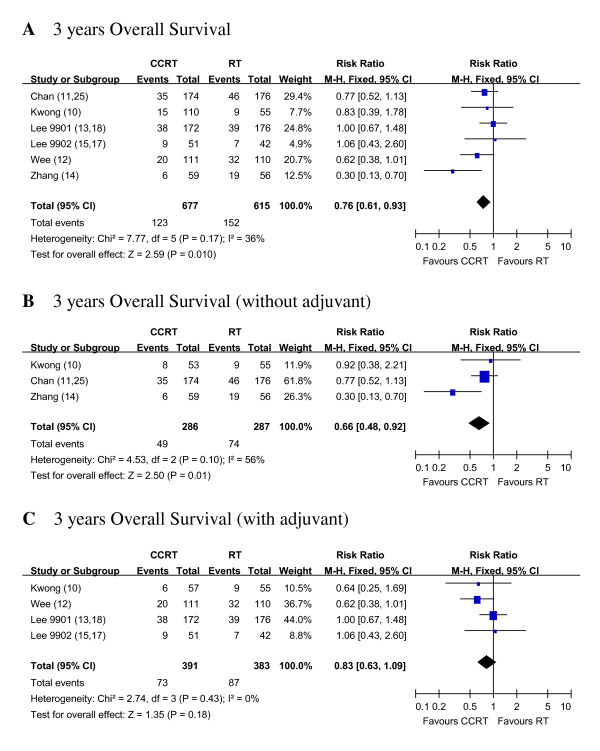
**Three years Overall Survival of RT alone vs. CCRT with and/or without AC**. Forest plots of the risk ratio (RR) of the 3 years OS with RT alone vs. pure CCRT and 3 years OS with RT alone vs. CCRT + AC. The estimate of the RR of each individual trial corresponds to the middle of squares and the horizontal line gives 95% CI. The closed diamond shows the overall RR with its 95%CI. RR less than 1 indicated improved survival for pure CCRT with or without AC compared with RT alone. Kwong_AB: In Kwong's trial, for subgroup analysis with pure CCRT vs. RT, group A and B were compared. Kwong_AD: In Kwong's trial, for subgroup analysis with adjuvant groups, A and D were compared.

For the second part of the analysis, we found that CCRT without AC was better than RT alone for 3 years OS. CCRT vs. RT showed RR of 0.66 (95% CI, 0.48 to 0.92) for 3 years OS, likewise CCRT+AC vs. RT showed RR of 0.83 (95% CI, 0.63 to 1.09) (Figure 3).

### Locoregional Recurrence

**Figure 4:**
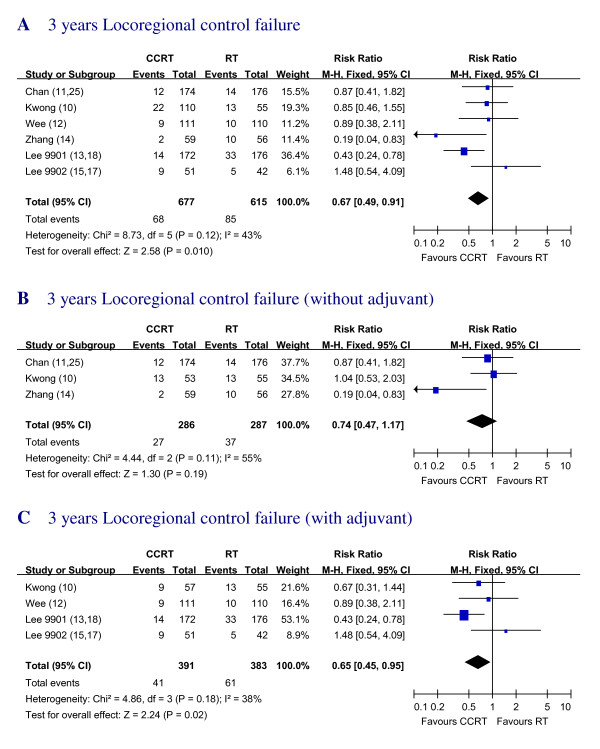
**Three years Locoregional control failure with RT alone vs. CCRT with and/or without AC **Forest plot of the risk ratio (RR) of 3 years locoregional control failure with RT alone versus CCRT with and/or without AC. The estimate of the RR of each individual trial corresponds to the middle of squares and the horizontal line gives 95% CI. The closed diamond shows the overall RR with its 95%CI. RR less than 1 indicated improved survival for the CCRT compared with RT alone. Kwong_AB: In Kwong's trial, for subgroup analysis with pure CCRT vs. RT, groups A and B were compared. Kwong_AD: In Kwong's trial, for subgroup analysis with adjuvant groups, A and D were compared.

Data regarding the absolute number of locoregional recurrence rate (LRR) for 3 years were available in the 6 studies [10-15]. A significant overall benefit in favor of the addition of chemotherapy was found with RR of 0.67 (95% CI, 0.49 to 0.91). The failure rate of locoregional control of CCRT group seems better compared to the RT alone group, the difference was significant (Figure. 4).

Additionally, the RR for 3 years LRR of CCRT vs. RT alone [10,11,14] and the LRR of CCRT+ AC vs. RT alone [10,12,13,15] were also calculated. LRR of CCRT without AC group had the RR of 0.74 (95% CI, 0.47 to 1.17) compared with RT alone. The group with CCRT plus AC showed the RR of 0.65 (95% CI, 0.45 to 0.95) (Figure.4).

### Distant Metastasis

**Figure 5:**
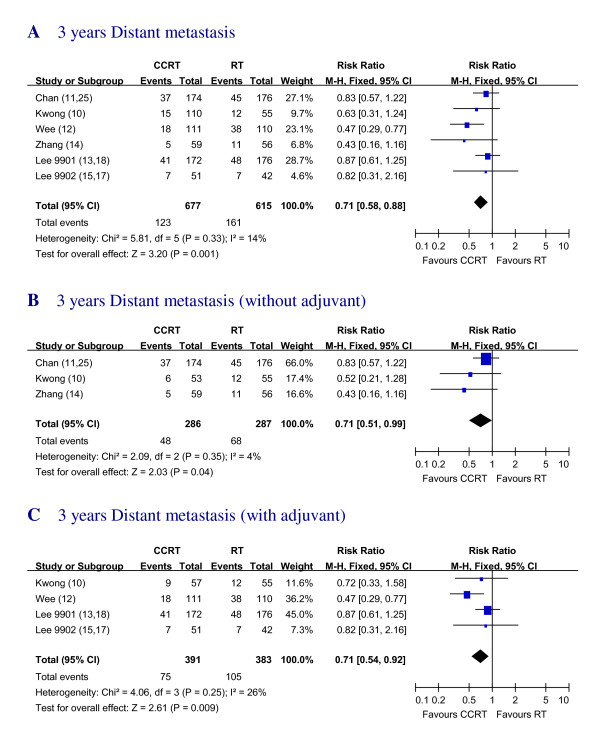
**Three years Distant metastasis rates with RT alone versus CCRT with and/or without AC**. Forest plot of the risk ratio (RR) for 3 years distant metastasis with RT alone versus CCRT with and/or without AC. The estimate of the RR of each individual trial corresponds to the middle of squares and the horizontal line gives 95% CI. The closed diamond shows the overall RR with its 95%CI. RR less than 1 indicated improved survival for the CCRT compared with RT alone. Kwong_AB: In Kwong's trial, for subgroup analysis with pure CCRT vs. RT, groups A and B were compared. Kwong_AD: In Kwong's trial, for subgroup analysis with adjuvant groups, A and D were compared.

Data regarding the absolute number of distant metastasis rate (DMR) in 3 years were provided in 6 trial reports [10-15]. A significant overall benefit in favor of the addition of chemotherapy was found with RR of 0.71 (95% CI, 0.58 to 0.88) (Figure. 5).

The RR for DMR of CCRT vs. RT alone [10,11,14] and DMR of CCRT +AC vs. RT alone [10,12,13,15] were also calculated. The CCRT group had the RR of 0.71 (95% CI, 0.51 to 0.99) compared with RT alone and the group with CCRT plus AC showed the RR of 0.71 (95% CI, 0.54 to 0.92) compared with RT alone (Figure. 5).

## Discussion

NPC is most common in Southern China and Southeast Asia, which accounts for the majority of NPC cases worldwide. The endemic type of NPC is generally different from western counterpart in pathological types, association with Epstein Barr Virus, natural history, and treatment. A meta-analysis which consists of patients purely from the endemic areas was long overdue.

This meta-analysis was designed to directly address the additional effect of chemotherapy concurrently combined with radiotherapy (CCRT) in endemic NPC population. These results suggested that the superior survival observed with CCRT compared with RT alone may be related significantly with improvement in the risk of distant metastasis.

Our analysis differed a little from the results of previous meta-analyses. The RR of 3 years OS (RR = 0.76) was larger than that detected in the other meta-analyses (RR = 0.16-0.60) [2,3,5], indicating that the benefit was smaller than anticipated (Table 2). The possible explanation for these differences is high proportion of patients with WHO type I histology in previous meta-analyses. In the current meta-analysis, almost all the cases were histologically proven NPC. More than 99.69% of these cases belonged to the WHO Grade II and Grade III subtypes. Only about 0.31% of the NPCs belonged to the WHO Grade I subtype. Whereas in other similar meta-analyses done in the past [2,3,5], significantly more percentage of patients with WHO Grade I type of tumor were included. As Grade I type of NPC is similar to squamous cell carcinoma of head and neck, which is more resistant to RT than Grade II and III type of NPC, patients with Grade I NPC subtypes may benefit mostly from CCRT [3,10]. But in endemic areas, most of the patients are Grade II and III type tumors, which are more sensitive to RT. In the current meta-analysis, we found that the contribution of survival benefit of CCRT mainly came from improvement of distant failure. This result also implied that NPCs in endemic areas are sensitive to RT. On the other hand, the RT methods are more aggressive in endemic areas than in other areas [1,10]. Therefore the margin of benefit potentially gained with additional chemotherapy may be reduced [12]. The finding that the RT alone group showed better 5-year survival rate in our analysis than in that reported by the intergroup study [1] (62% vs 21%) may also support this hypothesis. The current meta-analysis result indicated that CCRT was still the most effective treatment modality for the improvement of overall survival, but the exact magnitude of treatment effect of CCRT in endemic areas might be less than that previously reported by other meta-analyses. Further studies should be carried on exploring less toxic chemotherapy regimen for CCRT.

**Table 2:** Summary of the percentage of WHO type I tumors and outcome on overall survival

Study	Trials included	Patients with WHO type I tumors			OR/HR/RR		
		
		CCRT	RT	Total (%)	2 yrs	3 yrs	5 yrs
Huncharek et al [2] 2002*	Al-Sarraf et al [1]	17/78	19/69	36/147 **(24%)**	0.16	0.16	0.16

Langendijk et al [3] 2004^#^	Al-Sarraf et al [1]	17/78	19/69	48/781 **(6%)**	NA	NA	0.48
	Lin et al [8]	3/141	6/143				
	Chan et al [25]	2/174	1/176				

Baujat et al [5] 2006^#^	Al-Sarraf et al [1]	17/78	19/69	41/716 **(6%)**	NA	NA	0.60
	Chan et al [11]	2/174	1/176				
	Kwong et al [10] a). Concurrent b). Conc. +AC	1/53 0/57	1/55 0/54				

Our study 2010^§^	Kwong et al [10]	1/110	1/55	5/1608 **(0.31%)**	0.63	0.76	0.74
	Chan et al [11]	2/174	1/176				
	Wee et al [12]	0/111	0/110				
	Lee et al [13,18]	0/172	0/176				
	Zhang et al [14]	0/59	0/56				
	Lee et al [15,17]	0/51	0/42				
	Chen et al [16]	0/158	0/158				

Abbreviations: OR = Odds Ratio, HR = Hazard Ratio, RR = Relative risk (*used OR, ^#^used HR,^§^used RR in the analysis), NA = Not Available

In this analysis, we also tried to find if there is any additional benefit for the patients receiving CCRT plus some kind of AC. Sub-group analyses showed that both locoregional recurrences and distant failure were improved in CCRT+AC arms compared with RT alone. Pooling the results of these studies, the RR of death with CCRT+AC was not significant (figure 3, P = 0.18). This lack of survival difference might be due to increased mortality related to the toxicities of chemotherapy, and possibly successful salvage after relapse. Increase of non-cancer deaths due to treatment-related, incidental, and unknown causes might have also narrowed the actual magnitude of survival gain [13,15,17,18]. The role of adjuvant chemotherapy remains to be addressed by additional studies.

There was an indication from a previous trial by Lin et al [9] that the benefit of AC was associated with so called high-risk patients who met at least one of the following criteria: (1) nodal size >6 cm, (2) supraclavicular node metastases, (3) 1992 AJCC stage T4N2, (4) multiple neck node metastases with 1 node >4 cm. As our analysis did not include enough individual data, we could not analyze the effect of CCRT and AC vs. RT alone for the high-risk patients. We recommend that the future trials should be focused on the high risk patients.

One of the shortcomings of our meta-analysis is that all information came from published data instead of individual patient data, which might result in two sources of bias - publication bias and selection bias. We used funnel plot to estimate the publication bias. If the funnel plot is not symmetrical or not integrated then it suggests that the result may be biased. So we tested it by using the linear regression model proposed by Egger [23,24]. In our studies the funnel plot was symmetrical, suggesting that publication bias was not significant. To avoid the selection bias, two independent investigators reviewed the publications and extracted the data. The heterogeneity between the individual studies was also evaluated.

This meta-analysis was performed in geographic areas where NPC is endemic [10-18]. It still remained unclear whether the results obtained from trials performed in these endemic areas could be extrapolated to non-endemic areas.

## Conclusions

In conclusion, our meta-analysis based on published trials in endemic areas showed that the CCRT was the most effective treatment modality for the improvement of overall survival in locally advanced NPC. However, the relative benefit of CCRT in endemic population might be very different from previously published meta-analyses. In the future, treatment of NPC should be individualized, according to recognized prognostic factors, while recognizing the results of randomized trials of induction and concurrent CCRT.

## Competing interests

The authors declare that they have no competing interests.

## Authors' contributions

LZ, YG, YJH and CZ designed this study and contributed substantially to the design of the search strategy. YG and YJH developed the study protocol. YG and BG searched and selected the trials and extracted data. YG performed the analysis and interpreted the data. YG and BG wrote the manuscript. LZ and CZ critically reviewed the manuscript. HMH, QL, YZ and ZZG participated in the data extraction and critically revised it. YJH proofread the final version. All authors read and approved the final manuscript.

## Pre-publication history

The pre-publication history for this paper can be accessed here:

http://www.biomedcentral.com/1471-2407/10/558/prepub
